# Double-Network Tough Hydrogels: A Brief Review on Achievements and Challenges

**DOI:** 10.3390/gels8040247

**Published:** 2022-04-18

**Authors:** Hai Xin

**Affiliations:** ARC Centre of Excellence for Electromaterials Science, Intelligent Polymer Research Institute, Innovation Campus, University of Wollongong, Squires Way, North Wollongong, NSW 2522, Australia; thisflow@hotmail.com or hx936@uowmail.edu.au

**Keywords:** double network, toughness, stress relaxation, recoverability, 3D printing

## Abstract

This brief review attempts to summarize research advances in the mechanical toughness and structures of double-network (DN) hydrogels. The focus is to provide a critical and concise discussion on the toughening mechanisms, damage recoverability, stress relaxation, and biomedical applications of tough DN hydrogel systems. Both conventional DN hydrogel with two covalently cross-linked networks and novel DN systems consisting of physical and reversible cross-links are discussed and compared. Covalently cross-linked hydrogels are tough but damage-irreversible. Physically cross-linked hydrogels are damage-recoverable but exhibit mechanical instability, as reflected by stress relaxation tests. This remains one significant challenge to be addressed by future research studies to realize the load-sustaining applications proposed for tough hydrogels. With their special structure and superior mechanical properties, DN hydrogels have great potential for biomedical applications, and many DN systems are now fabricated with 3D printing techniques.

## 1. Introduction

Hydrogels are three-dimensional polymeric networks with super water-absorbing capabilities. Despite a large water content, hydrogels exhibit non-flowability and behave like a solid [1]. Since many hydrogels are prepared with biopolymers or biocompatible synthetic polymers, those hydrogels are proposed to have great potential for biomedical applications such as drug delivery, tissue regeneration, bio-scaffolds, artificial muscles, and bone replacement [2,3,4]. Nonetheless, many conventional hydrogels are mechanically weak and can be easily broken by an external force. The poor mechanical properties have substantially limited the applications of hydrogels, and how to enhance the mechanical performance of hydrogels becomes the most challenging question for gel researchers. The advent of tough hydrogels provides encouraging solutions for this problem because those hydrogel systems exhibit large fracture energy (from 10^2^ to 10^4^ J/m^2^) and super stretchability (9 times its original length), in addition to large water content and biocompatibility [5,6,7,8]. Thus, the tough hydrogels have the great potential to realize the above-mentioned biomedical and biomechanical applications. Several tough hydrogel systems have been reported including double-network hydrogels [5], clay–polymer nanocomposite hydrogels [6], hydrogen-bonded polyurethane hydrogels [9], and slide-ring hydrogels [10]. Among these reported tough hydrogels, double-network (DN) hydrogel demonstrates high fracture toughness and unique network topologies [5]. Since it was reported for the first time, it has attracted intense research interest to reveal the structural characteristics and toughening mechanisms of DN hydrogels. These research discoveries provide critical knowledge to improve the design and synthesis of existing DN hydrogels but also inspire materials scientists to develop novel hydrogel systems [11,12]. Even though DN hydrogels are interpenetrating networks where the two polymeric components are sequentially synthesized and infiltrated to form the overall system, their high fracture toughness considerably exceeds the traditional interpenetrating network hydrogels. This is attributed to many structural parameters—namely, swelling ratio, cross-linking density, ratio of the two hydrogel components, and molecular weight distribution of the first network polymer [12,13,14,15].

It has been almost 20 years since Gong et al. reported the first generation of DN hydrogels [5,16]. Tremendous research efforts have been made to study and improve this hydrogel system. It is very necessary to summarize the past research milestones pertaining the fracture energy, toughening mechanisms, potential shortcomings, and possible solutions for DN hydrogels. The present review aims to contribute a critical and brief analysis of this body of research. It provides fundamental background information to gel researchers to understand the fracture process of DN hydrogels and gives critical clues on the future development of tough soft materials for load-bearing applications. The major purpose of the present study is to give a concise and conceptual discussion on the fracture mechanisms and damage processes of DN hydrogels instead of making a comprehensive list of miscellaneous DN systems reported by various research groups.

## 2. Toughness and Damage Process of Covalently Cross-linked DN Hydrogels

The first double-network (DN) hydrogel system was reported in 2003 by Gong et al. DN Hydrogel systems exhibited remarkable mechanical strength of around 15 MPa, compared to single-network (SN) hydrogels prepared by the individual component of the corresponding DN hydrogels. Even though those SN hydrogels were smashed under compression stress, DN hydrogels were able to sustain a high level of mechanical stress, without observable damage [5]. Following this research article, in a series of research publications from Gong’s group, high fracture energy of DN hydrogels, was reported in a tearing test. In the test, the hydrogel samples were cut into a trouser shape, and the two arms of the trouser were pulled apart at a stable straining rate to allow the propagation of the pre-cut notch. The fracture energy was defined and calculated as the amount of energy dissipation per unit area during the crack development [15,17,18,19].

The DN hydrogels reported by Gong’s group were interpenetrating networks. The two-component networks were synthesized sequentially with the second network monomers absorbed and polymerized inside the first network. Two polymer networks were synthesized with free-radical polymerizations and cross-linked covalently. Therefore, the resultant DN hydrogels possessed significant network heterogeneity in the length of the polymeric strand between two adjacent cross-linking points [14,20], as reflected by the wide distribution of the molecular weight of the cross-linked polymer strand [13]. As reported, tough DN hydrogels were only obtained when the first network was tightly cross-linked, and the second network was sparsely cross-linked. Another important parameter was the molar ratio of the second network polymer to the first network. Such a ratio should be large to ensure the second network is the major component of the overall DN system [5,21].

These two parameters underlie the understanding of the damage process of DN hydrogels. As shown in successive load–unload tests, the DN hydrogel was stretched to a set strain and relaxed to its original length before it was re-elongated to the same or larger strain [14]. Once the set strain was above a threshold, significant hysteresis between stretching and retracting curves was observed. However, the reloading curve in the subsequent cycle followed the same path as the unloading curve in the previous cycle, showing irreversible energy consumption [13]. This phenomenon is called the Mullins effect, which demonstrates network damage of the tested materials [13,14,22]. When Gent’s model [23] was applied to fit the unloading curves of each test cycle, it was found that the fitted shear modulus kept decreasing, while the J_m_ values increased as a function of the extension ratios, calculated as the ratio of the current sample length to its original length. This is because the shear modulus represents the density of cross-linked polymer chains, and J_m_ represents the contour length of the remaining chains [23]. The load–unload test results clearly elucidate the damage process of DN hydrogels [13,14]. When the hydrogel sample is deformed, the shortest network strands caused by high cross-linking density in the first network are fully stretched and then broken to absorb the fracture energy, followed by the stretching and damage of the next-shortest chains at increased strains. It is the sequential breakage from the shorter to longer polymeric chains that dissipates the fracture energy and contributes to the high toughness reported for the DN hydrogels [14,24,25,26]. The damaged first-network polymer is then supported and stabilized by the loosely cross-linked second network, which prevents the catastrophic failure of the overall DN system. This is why the second network is required to be the major component of the DN system since it functions as a damage stabilizer [24,26].

## 3. Lake–Thomas Toughness of Covalently Cross-Linked DN Hydrogels

As introduced above, the most important parameter to creating tough DN hydrogels is the high cross-linking density of the first brittle network, which leads to a large number of short cross-linked polymer strands in the first network. The breakage of those short strands leads to the decrease in elastic modulus of DN systems and dissipates the fracture energy to make a DN hydrogel tough. The second network stops the catastrophic failure [2,20,26]. Based on this damage process, the Lake–Thomas theory [27] has been utilized to quantitatively estimate the correlation between DN fracture toughness and first network cross-linking densities.

**Figure 1:**
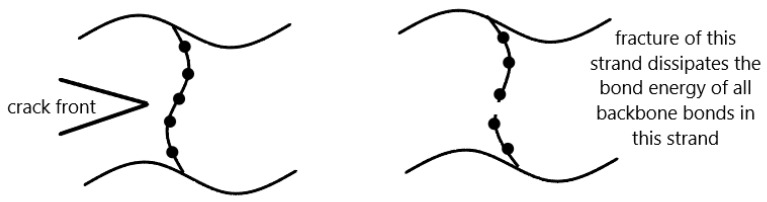
Schematic of Lake–Thomas theory. The black dots represent the carbon atoms, while the lines connecting two adjacent black dots represent the carbon–carbon bonds in the polymer strand. When the crack propagates and breaks one carbon–carbon bond, the bond energy of all backbone bonds will be dissipated. The fracture toughness of the broken strand is enhanced by the number of backbone bonds in the strand.

As shown in Figure 1, the original Lake–Thomas theory indicates that when one carbon–carbon bond in a cross-linked polymer strand is broken, the bond energy of all carbon–carbon bonds in this strand will be dissipated. The fracture toughness of the strand is enhanced by the total number of carbon–carbon bonds. The longer the strand is, the more backbone bonds there are, and the tougher it is [27].

In the case of covalently cross-linked DN hydrogels, the first network heterogeneity creates many short network strands, and those strands start to fracture progressively during the crack propagation, which is the major contributor to the dissipation of the fracture energy in a DN system [24]. According to Lake and Thomas [27], the fracture toughness of the first network per unit area of crack propagation can be calculated as

(1) G = ГnU

where G is the fracture energy of the first network (J/m^2^), Г is the areal density of cross-linked first network strands across the plane of the crack (mol/m^2^), n represents the average number of backbone bonds of network strands, and U is the covalent dissociation energy of each carbon–carbon bond (J/mol).

**Figure 2:**
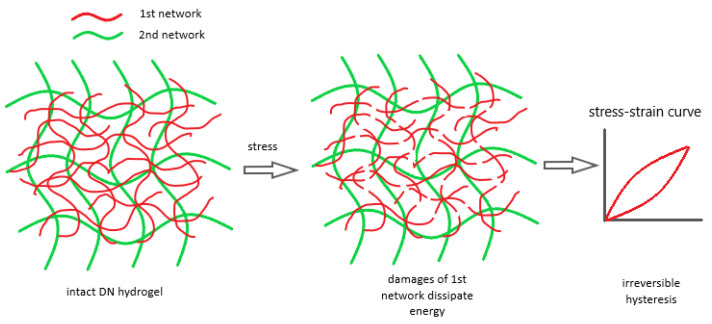
Damage process of DN hydrogels. The multiple damages in the 1st network dissipate large amounts of energy and lead to irreversible hysteresis in load–unload tests.

It is clear that G is determined by two factors: the areal density of cross-linked network strands across the crack plane (Г) and average backbone numbers in each strand (n). These two are competing factors, since increasing the cross-linker density increases Г but reduces n. As U in the equation shown above is a constant, the final toughness of the first network is directly associated with the product of Г and n. Research studies have managed to quantify the effects of cross-linker concentration on the swelling ratios and these two factors. It has been indicated that the overall concentration of backbone bonds in the first network that contributes to the energy dissipation during the crack development increased significantly when the first network cross-linker concentration increased from 2% to 4%. A linear correlation was found between the first network toughness and corresponding DN toughness [20]. This finding provides convincing evidence showcasing the role of the first network as a “sacrificial bond” for energy dissipator for the mechanical toughness of DN hydrogels [20,28]. Based on a model that used log-normal probability distribution theory to estimate the chain fracture of the first network [25], the strand length distribution of the first network prepared with various cross-linker concentrations was calculated. The results showed that higher cross-linker concentration created a much narrower distribution concentrated in the short strand length. The breakage of a considerable number of short strands of the first network is the major contributor to the fracture toughness of the entire DN system [13]. Figure 2 below demonstrates the origin of fracture toughness of DN hydrogels. The densely cross-linked first network is stretched and fractured to absorb mechanical energy. This energy dissipation process can be explained by the Lake–Thomas theory. Meanwhile, the damaged first network is stabilized by the sparsely cross-linked second network, which prevents failure of the entire DN system.

## 4. Mechanical Recoverability of Physically Cross-Linked DN Hydrogels

DN hydrogels synthesized with two covalently cross-linked networks are mechanically tough. However, due to its toughness originating from the irreversible rupture of the backbone bonds in the first network, the toughness and damage are not recoverable [14]. This structural drawback inherently limits the DN hydrogels for many load-bearing applications such as artificial muscles and tissue substitutes, which require the candidates to be tough and self-healing [29]. One of the possible solutions for this issue is to prepare the tough DN hydrogels with physical cross-links such as ionic coordination [30]. The fracture of the physical bonds absorbs mechanical energy to improve the toughness, and the broken bonds are able to re-form to rebuild the structures of the materials [7,11,22,31].

**Figure 3:**
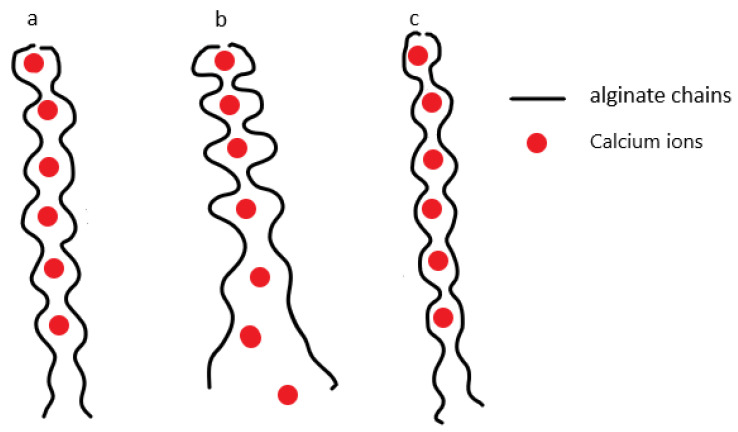
Schematic shows that (**a**) alginate chains are ionically chelated by Ca^2+^, and (**b**) under mechanical stress, the alginate–Ca^2+^ connections are pulled apart, and the fracture of these physical bonds dissipates mechanical energy; (**c**) upon removal of mechanical stress, the ionic cross-links are able to recover.

One important progress is ionic–covalent hybrid hydrogels that were prepared with alginate and polyacrylamide. These interpenetrating hydrogels have similar network structures to DN hydrogels, with alginate physically cross-linked by Ca^2+^ and polyacrylamide chemically cross-linked with covalent bonds. Since two types of cross-linkers are used, the hydrogel system is called a “hybrid”. The gel sample exhibited around high fracture energy of 9000 J/m^2^, assessed by pure-shear tests, and damage recoverability, as evidenced by the recovered hysteresis in the subsequent load–unload cycles [7]. The observed mechanical recoverability was attributed to the physical bonds between the Ca^2+^ and alginate chains, which behaved like an “egg-box” or “zip” [32]. Under mechanical forces, the alginate–Ca^2+^ connections are pulled apart to unzip, and this process absorbs the mechanical energy. Once the forces are removed, the ionic bonds between the alginate and free Ca^2+^ are reconstructed, exhibiting damage reformation. Figure 3 illustrates a schematic to show this reversible damage [33,34]. Gong et al. prepared the hydrogels based on polyampholytes, which feature randomly dispersed cationic and anionic groups. The ionic interactions between those oppositely charged groups exhibited a wide distribution of bond strength in which strong bonds function like “chemical cross-linkers” for elasticity, and the weak interactions act as “sacrificial bonds” for energy dissipation and damage reformation [35].

Inspired by these ionic–covalent hybrid hydrogels, a number of physically cross-linked hydrogels have been reported, with greatly enhanced toughness. Hydrogels toughened by hydrogen bonding are widely reported, exhibiting toughness of up to 9 kJ/m^2^ [36,37,38,39]. In one study, a naturally occurring biopolymer, agar, was used as the first network and cross-linked by a hydrogen bond, while the second network was polyacrylamide and chemically cross-linked. The resultant hydrogel systems reached the toughness of more than 1 kJ/m^2^, and the toughness was positively associated with the agar concentration [40]. Amylopectin was applied to introduce the stronger hydrogen bonds into the polyacrylamide–poly(vinyl alcohol) DN hydrogels to reinforce the existing weak hydrogen bonds between polyacrylamide and poly(vinyl alcohol). Consequently, both bulk toughness and interface toughness (between the gel and non-porous glass substrate) were enhanced, and self-recovery was demonstrated by the reversible hysteresis in cyclic stretch–retraction curves [41]. Internetwork hydrogen bonds formed between polyacrylic acid and poly(N-isopropylacrylamide) were also reported to increase the gel’s stiffness to up to 226 MPa [42].

Ionic interaction and hydrogen bonds can be both applied to prepare a DN hydrogel. In one study, a copolymer of acrylamide and acrylic acid was synthesized as the second network, while agar was used as the first network. The sample gel was then soaked in Fe^3+^ solution to facilitate the formation of ionic coordination between the ions and carboxylic groups. The agar network was expected to be cross-linked with the hydrogen bonds. This hydrogel system was reported to be tough, damage-recoverable (95% recovered hysteresis after 20 min), and fatigue-resistant [43].

Host–guest interactions between β-cyclodextrins and ferrocene (Fc) and dynamic borate ester bonds were also harnessed to prepare DN hydrogels, which exhibited superior stretchability, mechanical strength, and self-healing properties. The conductivity of this DN system was improved by the addition of carbon nanotubes, and the resultant DN hydrogel showed the capability of sensing diverse human movements as a potential strain sensor device [44]. Meanwhile, DN hydrogel can also be fabricated based on the hydrophobic interactions to cross-link both component networks. In a DN system prepared with curdlan and polyacrylamide, the two polymers were cross-linked with hydrophobic interactions, and the overall DN system was mechanically strong and demonstrated more than 90% toughness recovery once it was stored at 95 °C for 4 h [45].

## 5. Time-Dependence and Mechanical Instability of Physically Cross-Linked Hydrogels

**Figure 4:**
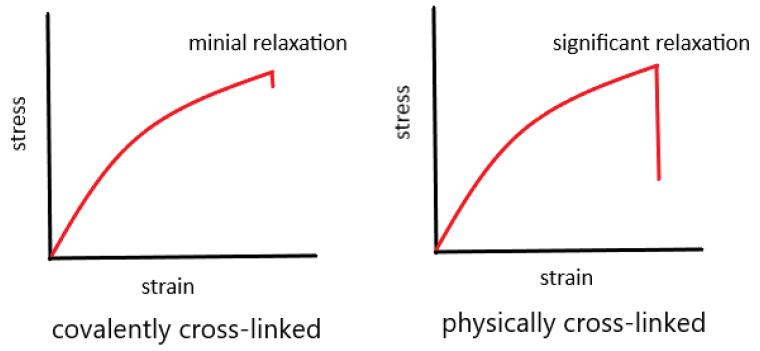
Schematics of stress relaxation tests for covalently cross-linked and physically cross-linked hydrogels. In the test, the hydrogel samples are stretched to a set strain and held to observe if the stress decays as a function of time. Physically cross-linked hydrogels exhibit substantial stress relaxation (mechanical instability). Covalently cross-linked hydrogels demonstrate small stress relaxation.

Physical cross-links, as discussed in the previous section, are used as sacrificial bonds to absorb the mechanical energy during the fracture process [46]. One inherent issue is that the fracture of these physical bonds is rate-dependent. When stretched at various straining rates, gel samples exhibit increased fracture energy with the increase in straining rates [47]. More importantly, in the stress relaxation tests in which the gels consisting of ionically cross-linked alginate are stretched and held at a set strain, the stress decayed with time. These results obviously suggest that the mechanical properties of those physically cross-linked hydrogels are mechanically unstable. Their stress and toughness are time-dependent and will relax and decay with time [48,49,50,51]. This issue may greatly limit their applications as load-bearing biomedical devices such as drug delivery and artificial muscles [4,52,53]. Thus, one of the key challenges facing tough hydrogel scientists is to improve both mechanical toughness and stability simultaneously. In Figure 4 below, schematics of stress relaxation for covalently cross-linked and physically cross-linked hydrogels are presented. Clearly, physically cross-linked hydrogels exhibit stress relaxation, which suggests that they are unable to sustain the stress over a long period of time, even though they are damage-recoverable, which is advantageous over conventional covalently cross-linked DN hydrogels. On the other hand, chemically cross-linked hydrogels exhibit minimal stress relaxation and mechanical stability, due to their covalent bonds. The disadvantage of chemical hydrogels is their irreversible structural damage.

Some efforts have been made to understand how to tune the relaxation behavior of alginate-based hydrogels. Sparks et al. [49] prepared interpenetrating network hydrogels based on alginate and polyacrylamide. It was found that reducing the cross-linking density of the polyacrylamide network increased the stress relaxation of the entire hydrogel system. This observation was attributed to the increased free movement of polyacrylamide chains as a result of reduced cross-linker concentration. The swelling ratio was also found important. The polymer chains in the swollen state were more stretched, and their free motion was restricted, thus being unable to induce stress relaxation. However, the increased swelling ratio also decreased the stiffness of the sample hydrogels [49]. In another study, polyethylene glycol chains were chemically grafted onto alginate chains cross-linked by Ca^2+^. The stress relaxation of the hydrogel was positively correlated to the amount of grafted polyethylene glycols, which increased the interactions between the alginate chains, leading to an increase in relaxation behaviors [54]. Future investigations need to address how the balance between mechanical toughness and stress relaxation can be reached, and how the ionically cross-linked alginate can be modulated to reduce its stress relaxation or creep behaviors.

Some tough hydrogels exhibiting fast recovery, mechanical resilience, and fatigue resistance have been reported [8,29,55]. Burdick et al. [56] recently prepared injectable DN hydrogels. In the gel system, the methacrylated hyaluronic acid chains were modified with β-cyclodextrin (CD) and adamantane, respectively, and then mixed to form guest–host connections. This physically cross-linked network was further incorporated into a chemically cross-linked second network based on methacrylated hyaluronic acid. The resulting DN hydrogels demonstrated fast self-healing and no Mullins hysteresis during repetitive load–unload tests. In one latest research attempt, Ito et al. [8] synthesized slide-ring hydrogels [10] wherein polyethylene glycol chains were threaded into hydroxypropyl-a-cyclodextrin (CD) rings, and the CD rings were chemically connected to form the hydrogel network. The obtained hydrogel system exhibited reversible crystallization of PEG chains and instant damage recovery during cyclic load–unload tests.

Even though the tough hydrogels with self-healing and rapid recovery properties may offer a possible solution for conventional DN systems with Mullins hysteresis. To the best of the author’s knowledge, few research articles have been performed in which stress-relaxation measurements were used to verify their mechanical stability. This issue remains an unresolved question and needs to be addressed in the future.

## 6. Biomedical Applications of Tough DN Hydrogels

Tough DN hydrogels have been proposed and applied in diverse biomedical applications, including tissue engineering, drug delivery, wound dressing, biosensors, etc. [3]. Recently, Gong et al. have reported self-growing DN hydrogels. Responding to the repeating mechanical stimuli, this hydrogel system is able to transduce mechanical signals to chemical reactions, which enables the self-strengthening of the gel network. Due to this structural characteristic, the reported DN hydrogel may be promising for applications in artificial muscles and soft robotics [57].

In tissue engineering, the application of tough DN hydrogels as tissue scaffolds has been proposed and attempted. Facilitated with extrusion-based 3D printing, alginate–acrylamide ink was printed, and the acrylamide was in situ UV cross-linked to form polyacrylamide. The obtained hydrogel can be further reinforced by CaCl_2_ solution immersion. The reported fabrication process has been proposed to have applications in cell scaffold preparation [58]. The 3D printing inks were also prepared by the blend of alginate, poly(ethylene glycol diacrylate) (PEGDA), and laponite XLS was applied to optimize the ink viscosity. This ink can be printed into complex shapes and encapsulated with both bone marrow-derived mesenchymal stem cells and human embryonic kidney 293 cells such that it offers an encouraging solution for tissue engineering [59]. Both chitosan and alginate are widely used biopolymers for tissue growth scaffolds. These two polymers were mixed in the presence of hydrochloride acid. The protonated chitosan formed an electrostatic complexion with negatively charged alginate. The gel solution was 3D-printable and exhibited the proliferation of seeded human adipose stem cells [60]. Double-network granular hydrogels (DNGH) have also been reported. Different from conventional DN systems, DNGH hydrogels were prepared by soaking poly(AMPS) microgels in acrylamide solutions containing the cross-linker. Then, the mixture was 3D-printed and UV-cured to form a percolating network. The resultant DNGH hydrogel exhibited mechanical strength of around 1.3 MPa and increased mechanical toughness, compared with its component single network hydrogels [61]. In another study, highly cross-linked microgels were swollen in hydrogel precursors as the second network, which contained monomers, cross-linker, and initiator. Those microgels were found to be able to adjust the rheological properties of the mixture ink. The ink was then 3D-printed to form a loosely cross-linked second network, which was interpenetrating with the microgel network. The obtained 3D-printable double-network hydrogels demonstrated superior fracture toughness in pure shear tests and were proposed to have biomedical applications in tissue engineering and soft robotics [62].

Drug delivery is another area where tough DN hydrogels are considered very useful. Tough and smart DN hydrogels with superior load-bearing performance and stimuli responsiveness can be injected into the organs and tissues, which may undergo extensive physical movement during daily activities. The toughness of DN hydrogels overcomes the shortcomings of many conventional hydrogel systems for this application [4]. Chitosan and sodium alginate were used to prepare the DN hydrogels to deliver the drug, and this DN system exhibited a sustained and pH-tunable in vitro drug release profile [63]. In another DN system [64], the integration of a polyacrylamide network and polyacrylic acid modified by cyclodextrin was capable of delivering the peptide with an adjustable release profile. The interaction between the cyclodextrin and the peptide was reversible, and it played a critical role in tuning the release profiles without any impact on the mechanical properties of the host hydrogels. Rodell et al. [56] prepared DN hydrogels based on methacrylated hyaluronic acid. The DN system featured supramolecular guest-host association and covalent crosslinking. The reported DN system was injectable and cytocompatible, and facilitated cell proliferation.

The DN hydrogels’ capability to sustain a large degree of mechanical strains endow their applications as strain sensors to detect and measure human motions. In one example, DN hydrogels were synthesized with self-recoverable host–guest interaction and dynamic borax ester bonds. The resultant DN system was further reinforced with carbon nanotubes to enhance its electroconductivity and enable the detection of various human movements from fingers, wrist, elbow, knees, blink, etc. [44]. Another example was to utilize DN hydrogels based on polyacrylamide and poly(sodium acrylate) as the first network, poly(vinyl alcohol) as the second network, and sodium chloride to increase ion conductivity. Such a DN hydrogel exhibited good conductivity, large stretchability, and superior response to strain and fatigue resistance, making this DN hydrogel an encouraging candidate for strain sensors in wearable devices [65]. In the latest attempt, a DN hydrogel was prepared with a hydrogen-bonded agar network and covalently cross-linked polyacrylic acid. The polyacrylic acid network was further cross-linked with reversible coordination with Fe^3+^. The reported DN hydrogel demonstrated damage recoverability and high sensitivity to the strain [66].

Last but not least, DN hydrogel is also realizing its application in wound dressing, owing to its remarkable mechanical toughness, self-healing properties, and biocompatibility. For example, a DN hydrogel was fabricated with physically cross-linked gelatin and covalently cross-linked poly(γ-glutamic acid). The obtained DN hydrogels exhibited high compression strength at 38 MPa and also showed the ability to support cell adhesion and proliferation. Its effects on the healing process of a full-thickness skin wound defect were observed in rat models [67]. In order to treat diabetics foot ulcers, a research group [68] fabricated DN hydrogels with gelatin, polyacrylamide, and ε-polylysine, which demonstrated remarkable stretchability and proper moduli to suit skin dressings. The obtained DN system also exhibited superior abilities to promote collagen deposition and angiogenesis and suppress bacteria growth and infection.

## 7. Conclusions and Future Perspectives

The present review summarizes the research progress on mechanical toughness, damage recovery, stress relaxation, and biomedical applications of double-network hydrogels. Since it was reported by Gong et al. in 2003, DN hydrogel has captured intense interest owing to its special network topology and superior toughness. Various synthetic and natural polymers have been used in DN hydrogel preparation, and its large fracture energy has been found to be directly correlated to the breakage of a highly cross-linked first network. The damaged first network is stabilized by the loosely cross-linked second network, which prevents the catastrophic failure of the entire DN system. Inspired by conventional DN hydrogels in which both networks are covalently cross-linked and exhibit Mullins hysteresis, many physically cross-linked tough hydrogels have been synthesized. One example is ionic–covalent hybrid hydrogels in which the first alginate network is chelated by Ca^2+^, while the second polyacrylamide acts as a damage stabilizer. Although these hydrogels demonstrate damage recoverability, their intrinsic drawback is stress relaxation and time-dependent toughness. This may limit their load-sustaining applications such as artificial muscles and substitutes for damaged tissues. It is an urgent and unresolved task to achieve both mechanical toughness and stress stability simultaneously. Meanwhile, with the rapid development of 3D printing techniques, many 3D-printable DN hydrogels have been reported, with superior shape fidelity, mechanical properties, and cytocompatibility. These hydrogels can promote cell proliferation, which underlay the future applications of DN hydrogels in tissue engineering and drug delivery.

The most significant challenge that remains unresolved is the mechanical instability of many physically cross-linked hydrogels. Due to their physical nature, these hydrogels are damage-reversible and therefore superior to the conventional DN hydrogels, showing irreversible structural fracture. However, physical hydrogels also exhibit the inability to sustain stress for a long period of time, as reflected in stress relaxation time and rate-dependent toughness. It is a crucial task to address this issue and accomplish toughness and stability in one system to mimic real muscles or human tissues that are both mechanically tough and stable. This challenge has not been addressed properly, and more tests need to be carried out to characterize the mechanical stability of any newly developed tough hydrogel system, in order to realize the load-bearing applications of tough hydrogels.
